# Supplementary material for: Using peptide-exchange systems to interrogate peptide-specific KIR binding to HLA Class I
Source: Discov Immunol. 2026 May 25;5(1):kyag010. doi: 10.1093/discim/kyag010 (PMC13262528; doi:10.1093/discim/kyag010)
Supplement: kyag010_Supplementary_Data [file kyag010_supplementary_data.docx]

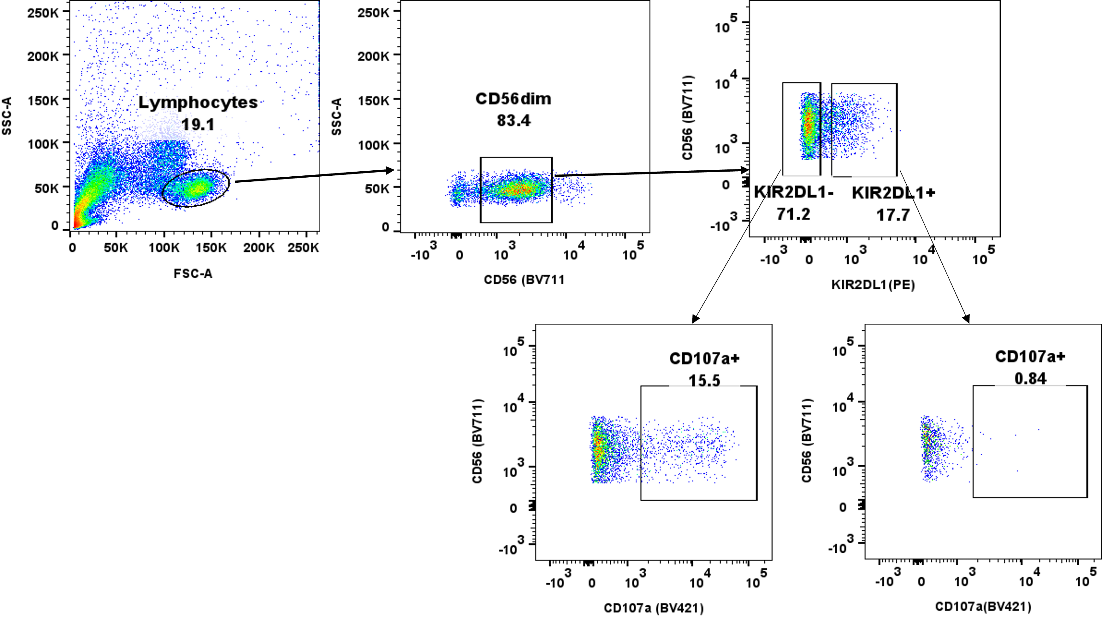


Supplementary figure 1: Full gating strategy for figure 3B. Cells were first gated on lymphocytes, followed by CD56^Dim^ (BV711) cells, and then on KIR2DL1^-^ and KIR2DL1^+^ (PE) cells. Cells were subsequently gated and analysed for CD107a (BV421) expression.
